# Hysteroscopic Resection for Missed Abortion: Feasibility, Operative Technique and Potential Benefit Compared to Curettage

**DOI:** 10.3389/fsurg.2020.00064

**Published:** 2020-09-08

**Authors:** Matthieu de Codt, Claire Balza, Pascale Jadoul, Patrice Forget, Jean-Luc Squifflet, Pierre Bernard, Mathieu Luyckx

**Affiliations:** ^1^Department of Gynecology and Andrology, Cliniques Universitaires Saint-Luc, Brussels, Belgium; ^2^Clinical Chair in Anaesthesia, University of Aberdeen, Honorary Consultant, NHS Grampian, Aberdeen, United Kingdom; ^3^Department of Obstetrics, Cliniques Universitaires Saint-Luc, Brussels, Belgium

**Keywords:** missed abortion, management, curettage, hysteroscopic resection, reproductive outcomes

## Abstract

**Objective:** To evaluate the feasibility of hysteroscopic resection (HsR) for primary surgical management of missed abortion. Reproductive outcomes and potential benefit of this technique will be compared to traditional dilatation and curettage (D&C).

**Design:** Retrospective cohort study in two Departments (Gynecology and Obstetrics) of a tertiary medical care center (Canadian Task Force classification II-2).

**Patients:** Women with first trimester missed abortion.

**Intervention:** Two techniques were used for the management of missed abortion: ultrasound-guided dilatation and curettage (D&C) and hysteroscopic resection (HsR).

**Results:** We evaluated 358 patients who underwent primary surgical removal of missed abortion. Hundred seventy three patients have been treated by D&C and 185 underwent HsR. In the HsR group, 110 patients (59.5%) have obtained their pregnancy with *in vitro* fertilization (IVF) vs. 7 patients (4.0%) in the D&C group which make the HsR population hypofertile in comparison to the D&C population. The intra- and post-operative complication rates are low and comparable. Intrauterine anomalies were diagnosed during the HsR in 10 patients (5.4%) and could be investigated after the intervention as a possible cause of miscarriage. Because of the difference in term of fertility, the reproductive outcomes have been analyzed by multivariate analysis. The hazard ratio of pregnancy at 6 months, adjusted to the factor IVF for D&C compared to HsR is 0.69 [0.49–0.96] (*p* = 0.026). That could represent a significant benefit in the particular population followed in IVF, but regarding the retrospective analysis, and the very different population in the two groups, it doesn't allow us to draw any evidence based conclusion.

**Conclusion:** Hysteroscopic resection is a feasible and safe procedure for the management of missed abortion that could increase the diagnosis of uterine abnormalities. With all the limitation of the design of our study, our data seems to show a trend to a potential benefit in term of reproductive outcomes for hypofertile patient undergoing IVF treatment.

## Introduction

Approximately 10–20% of first trimester pregnancies end up in a miscarriage (1). In some cases, the product of conception remains either in full (missed abortion) or in part (incomplete miscarriage) in the uterine cavity and is called “Retained product of conception” (2) (RPOC). They are classically managed using three different methods: expectant care, drug-based management and D&C. The patient generally considers the miscarriage as a failure and usually wants to get pregnant as soon as possible after this episode. Hence the frequency of this situation and the importance of preserving fertility, the three methods are frequently compared in the literature (1–3). The existing literature suggests that D&C allows a better clearing of the uterus when compared to the two other techniques (3) but it induces more intrauterine adhesions (4) (IUAs). The medical and expectant management sometimes require an emergency curettage for uncontrolled bleeding or abdominal pain (2). Despite these facts, the three methods show no difference in terms of infections (2) and fertility (5).

Up to now, D&C is the method of choice for surgical evacuation of residual trophoblastic tissue. Vacuum aspiration should be preferred to sharp curettage as it causes less pain and less blood loss (6). It is also complicated by fewer IUAs (7) which still occur in about 15% (8) and compromise the fertility of the patient. Some new opportunities have recently been developed to improve the outcome of surgical management of miscarriage. The ultrasound-guided curettage allows more accuracy in the resection of trophoblastic tissue and endometrium than blind D&C (9). The hysteroscopic resection (HsR) of RPOC is also a new alternative to D&C. A few studies (9–11) have already compared the two surgical techniques in case of post-operative or post-partum trophoblastic remnants. To our knowledge, there is no study comparing D&C to HsR in primary surgical management of missed abortion.

## Materials and Methods

After approval of the “Comité d'Ethique Hospitalo-Facultaire” (reference number: 2014/20MAI/254), we conducted a retrospective analysis of all the patients presenting a missed abortion managed by HsR between 2010 and 2014 in the department of Surgical Gynecology, IVF and Andrology of the Cliniques universitaires Saint-Luc, Brussels, Belgium. The data's from the operative report, early, and post-operative complication and reproductive outcomes were collected. In order to have a classical D&C arm to compare, we also collected the data's from the patients managed in the department of Obstetrics of Cliniques universitaires Saint-Luc that use systematically ultrasound guided D&C to manage missed abortion.

The research was made using Medical Explorer® software and Diamm®. Statistical analysis was performed using the Kolmogorov-Smirnov test to evaluate the data normality. Student and χ^2^ tests were used to study continuous and categorical variables. We performed a success of pregnancy analysis adjusted to the conception mode 6 months after the procedure (spontaneous vs. assisted) with a multivariable Cox regression model. *p* < 0.05 was considered statistically significant. All analyses were performed using STATISTICA software version 7 (Statsoft, Inc., 2004). This paper is reported following the “STrengthening the Reporting of OBservational studies in Epidemiology” (STROBE) statement (12).

### Operative Technique

- Ultrasound-guided curettage: the procedure is performed under general anesthesia. The cervix is dilated to Hegar no. 10 to allow introduction of a suction cannula. The trophoblastic tissue is then removed by vacuum aspiration with ultrasound guidance. Should significant bleeding occur, 10 IU of Syntocinon® would be administered intravenously.
- Hysteroscopic resection: the procedure is conducted under general anesthesia. The cervix is dilated to Hegar no. 10. Uterine distension is made by continuous glycine flow. Hystero-resectoscope is introduced with the loop to keep unstuck the trophoblastic sac by gentle motions without application of current. A polyp forceps is then inserted through the cervix to grab the free sac. Re-introduction of the hysteroscope to verify the emptiness of the uterus (supplemental video shows full length procedure). The remaining level of glycine is controlled at the end of the procedure to evaluate the resorption. Should significant bleeding occur, 10 IU of Syntocinon® would be administered intravenously.

## Results

Between 2010 and 2014, 173 patients in the D&C group and 185 in the HsR group were, respectively, managed in the Obstetrics and Surgical Gynecology, IVF and Andrology Departments of our hospital for missed abortion. The patient's data are summarized in Table 1. Demographics for both groups were similar in term of age and previous uterine surgery but the two populations were different in term of fertility: 110 patients (59.5%) in the HsR group has obtained their pregnancy by *in vitro* fertilization (IVF) vs. 7 patients (4.0%) in the D&C group. This difference was due to the general organization of the hospital. IVF service is part of the Gynecologic Department and then miscarriage after IVF are preferentially addressed to the surgeon from this Department, performing systematically HsR. The spontaneous miscarriage in the event of known intra uterine pregnancy, not from the IVF service, are systematically addressed to the Obstetrics Department. This could also explain the significant difference in gravidity, parity, gestational age at miscarriage, and drug based management (IVF patients usually prefer expectant management as it is easier psychologically than drug based management). Both interventions were performed by gynecologists or senior interns in gynecology. Intraoperative and post-operative complications are low and comparable in both groups as shown in Table 2 with a slightly increased risk of hemorrhage in the D&C group. There were no complication due to glycine resorption. Intrauterine anomalies were diagnosed during the HsR in 10 patients (5.4%) while this information is not available in regard to the D&C technique. There was no systematic control of the cavity after the procedure so we have no information regarding the potential trophoblastic remnants and IUA's after surgery.

**Table 1:** Characteristics of the patients undergoing either HsR or D&C for missed abortion (*n* = 358).

**Characteristics**	**HsR (*n* = 185)**	**D&C (*n* = 173)**	***p*-value**
Age (years; mean ± SD)	34.1 ± 5.6	34.2 ± 5.2	0.360
Gravidity (*n*; mean ± SD)	2.4 ± 1.6	3.0 ± 1.9	<0.001
Parity (*n*; mean ± SD)	0.6 ± 1.0	1.1 ± 1.1	<0.001
Previous uterine surgery	60 (32.4%)	56 (32.4%)	1.000
Mode of pregnancy			<0.001
IVF	110 (59.5%)	7 (4.0%)	
Spontaneous	75 (40.5%)	166 (96.0%)	
Gestational age at miscarriage (week ± SD)	9.1 ± 2.3	9.9 ± 2.4	0.004
Time delay before surgery (days ± SD)	11.3 ± 5.9	8.7 ± 5.6	0.004
Failure of prostaglandin treatment	17 (9.2%)	122 (70.5%)	<0.001

**Table 2:** Surgical characteristics and complications following HsR and D&C for missed abortion.

	**HsR (*n* = 185)**	**D&C (*n* = 173)**	***p*-value**
Intervention median time (min)	00:20	00:16	<0.001
Diagnosis of intrauterine anomaly	10 (5.4%)	^***^	
Polyp	2		
Submucosal fibroid	3		
Uterine septum	2		
IUAs	3		
**Intraoperative complication**			
Cervical tears	1	1	0.962
Perforation	1	0	0.33
Hemorrhage (>500cc)	0	4	0.037
**Post-operative complication**			
Due to glycine resorption	0	^***^	
Endometritis	2	1	0.601

*** *Non applicable*.

The post-operative fertility is presented in Table 3. Hundred sixty one patients in the D&C group and 131 in the HsR group wanted to be pregnant again. There were, respectively, 42 and 72 patients lost to follow-up (LTFU) in the HsR and D&C groups. There is no difference in the meantime of conception between the two groups. As previously reported, the use of assisted reproductive therapy is considerably higher in HsR than D&C group making the first one a “hypofertile” population. Hence this fact and the important rate of lost-to-follow-up, a multivariate Cox regression model, adjusted to the IVF factor, was performed and limited at 6 months to reduce the influence of the LTFU patients. The difference in pregnancy rate is significantly greater in the HsR group than in the D&C group with, respectively, 52/131 (39.7%) and 43/161 (26.7%) pregnancies at 6 months (*p* = 0.018). The hazard ratio at 6 months is 0.69 [0.49–0.96] (*p* = 0.026). With all the limitations of a retrospective analysis and the very different population in the two groups, our result seems to show an advantage in reproductive outcomes of HsR compared to D&C in a hypofertile group of patient who require IVF to obtain their pregnancies.

**Table 3:** Reproductive outcomes at 6 months after HsR or D&C for missed abortion.

	**HsR (*n* = 185)**	**D&C (*n* = 173)**	***p*-value**
Lost to follow-up at 6 months	42	72	<0.001
Patients with desire of pregnancy	131	161	<0.001
Overall pregnancy	52/131 (39.7%)	43/161 (26.7%)	0.018
Mean time to conception (months)	4.73	4.77	0.35

## Discussion

Residual trophoblastic tissue is a common complication of miscarriage. When a surgical management is needed, RPOC are usually treated by D&C. A significant complication of these interventions are IUAs which occur in ~15% for a single curettage (8) and 40% for repeated curettage (13) and compromise fertility. Since miscarriage usually occurs in patients desiring pregnancy, fertility is a key point in the management of miscarriage. As alternatives to blind D&C, ultrasound-guided curettage and hysteroscopic resection of RPOC have been developed in order to visualize the intervention in an attempt to minimize unnecessary trauma to the endometrium. There are 3 studies comparing D&C to HsR showing fewer IUAs (9), better pregnancy rate (9) and quicker post-operative pregnancy (9–11) in the HsR group for patients with post-operative or post-partum trophoblastic remnants. To our knowledge, this study is the first to explore HsR in primary surgical management of missed abortion and to evaluate reproductive outcomes in comparison with classical D&C technique.

We found similar rates of intra- and post-operative complications and there were no complication due to glycine resorption for which surgeons are wary of haemodilution (14). HsR seems to be as safe as D&C in the treatment of missed abortion. Furthermore, the HsR allowed the diagnosis of intrauterine anomalies in 5.4% of patients. Those were sometimes treated during the same intervention but were mostly investigated at distance of the miscarriage. This is particularly interesting knowing that intrauterine anomalies such as polyps, septum and fibroid can favor miscarriage.

Regarding the reproductive outcomes, sub analysis of our data (trying to minimize the differences between the two groups and reducing the impact of the importance of the LTFU) showed a hazard ratio of pregnancy at 6 month of 0.69 for the D&C compared to HsR. This technique could be a good choice for a hypofertile population needing IVF to obtain their pregnancy. It also rejoin the conclusion of Ozgur et al. in their study (15) which shows a six months recovery time needed to restore the reproductive capacity after D&C for patient followed in IVF.

The retrospective design, the number of patients lost-to-follow-up and the heterogeneity between the two groups are clearly limiting the strength of our conclusions but could, as all the research studies, be the first step to build more qualitative studies to evaluate the best surgical management for missed abortions.

In conclusion, the present study shows that hysteroscopic resection is feasible and as safe as D&C for the management of missed abortion. It seems to be associated with better pregnancy rate at 6 months in a hypofertile population requiring IVF. This exploratory analysis of HsR for the management of missed abortions is very encouraging and could be a step to design a prospective randomized trial over the surgical management of missed abortions.

## Data Availability Statement

All datasets presented in this study are included in the article/Supplementary Material.

## Author Contributions

MC is the author who has written the manuscript. The data were collected by MC and CB. PF did the statistical analysis. PJ, J-LS, and PB participated actively in drafting sections of the manuscript, editing, and approving the final submitted version. ML is the main supervisor of this work and he revised the final article. All authors contributed to the article and approved the submitted version.

## Conflict of Interest

The authors declare that the research was conducted in the absence of any commercial or financial relationships that could be construed as a potential conflict of interest.
